# P030: Role of unnecessary movement of patients in spread of C. difficile infection

**DOI:** 10.1186/2047-2994-2-S1-P30

**Published:** 2013-06-20

**Authors:** N Damani, S Wallace

**Affiliations:** 1 Southern Trust, Craigavon, UK

## Introduction

*C.difficile* associated diarrhoea (CDAD) has been recognised as the most common cause of hospital acquired diarrhoea. Among others the unnecessary movement of patients between wards is one of the major factors in the spread of CDAD. The aim of this study was to map the journey of all patients with CDAD during their inpatient stays over a 3 year period and assess the impact on CDAD spread.

## Methods

Every patient who was diagnosed with CDAD between January 2008 and December 2010 in our hospital was identified and their inpatient stay history was mapped using the electronic Patient Administration System. Cross infection was defined if two patients were in the same ward simultaneously and had the same ribotype. For cross infection purposes we used the 078 ribotype as a surrogate marker as this was the predominant strain within our hospital.

## Results

Retrospective analysis of all 078 ribotypes in our hospital showed that that majority of patients were admitted to the Medical Admission Unit for initial assessment and then transferred onward to other wards. From this we have identified 13 opportunities of possible cross infection among patients who had the same ribotype.

## Conclusion

The study supports the findings that among other factors, unnecessary movement of patients must be kept to a minimum to prevent spread of CDAD. As a result of this study, all unnecessary movements stopped throughout the hospital and all patients who had a previous history of CDAD are now directly admitted to a single room or admitted to a dedicated isolation ward. This change in practice, along with other infection control measures has resulted in a reduction in newly acquired CDAD cases from 1.0 (2009-10) to 0.3 (2010-11) cases per 10,000 beds days. Post-intervention we have also seen a substantial decline in CDAD cases caused by 078 ribotypes.

## Disclosure of interest

None declared

